# Circulating Hepatitis B Surface Antigen Particles Carry Hepatocellular microRNAs

**DOI:** 10.1371/journal.pone.0031952

**Published:** 2012-03-28

**Authors:** Luisa Novellino, Riccardo L. Rossi, Ferruccio Bonino, Daniela Cavallone, Sergio Abrignani, Massimiliano Pagani, Maurizia R. Brunetto

**Affiliations:** 1 Hepatology Unit and Liver Physiopathology Laboratory, University Hospital of Pisa, Pisa, Italy; 2 Department of Integrative Biology, Istituto Nazionale di Genetica Molecolare (INGM), Milan, Italy; 3 General Medicine Unit 2, Department of Internal Medicine, University Hospital of Pisa, Pisa, Italy; Yonsei University, Republic of Korea

## Abstract

Hepatitis B virus (HBV) produces high quantities of subviral surface antigen particles (HBsAg) which circulate in the blood outnumbering virions of about 1\10^3–6^ times. In individuals coinfected with the defective hepatitis Delta virus (HDV) the small HDV-RNA-genome and Delta antigen circulate as ribonucleoprotein complexes within HBsAg subviral particles. We addressed the question whether subviral HBsAg particles may carry in the same way cellular microRNAs (miRNAs) which are released into the bloodstream within different subcellular forms such as exosomes and microvescicles. Circulating HBsAg particles were isolated from sera of 11 HBsAg carriers by selective immunoprecipitation with monoclonal anti-HBs-IgG, total RNA was extracted and human miRNAs were screened by TaqMan real-time quantitative PCR Arrays. Thirty-nine human miRNAs were found to be significantly associated with the immunoprecipitated HBsAg, as determined by both comparative DDCT analysis and non-parametric tests (Mann-Whitney, p<0.05) with respect to controls. Moreover immunoprecipitated HBsAg particles contained Ago2 protein that could be revealed in ELISA only after 0.5% NP40. HBsAg associated miRNAs were liver-specific (most frequent = miR-27a, miR-30b, miR-122, miR-126 and miR-145) as well as immune regulatory (most frequent = miR-106b and miR-223). Computationally predicted target genes of HBsAg-associated miRNAs highlighted molecular pathways dealing with host-pathogen

The finding that HBsAg particles carry selective pools of hepatocellular miRNAs opens new avenues of research to disentangle the complex interactions between host and HBV and provides a non invasive tool to study the physiopathology of liver epigenetics.

## Introduction

Hepatitis B virus (HBV) is a non-cytopathic, hepatotropic virus with complex interactions with the host's immune system [1]–[2]. HBV engages the cellular machinery of infected hepatocytes for the assembly and release of double-shelled 42 nm complete virions and 20 nm subviral particles [1]. HBV produces extremely high quantities of hepatitis B surface antigen (HBsAg), the coating structure of both virions and defective particles which outnumber virions 10^3^–10^6^ times [1]. Moreover, in individuals coinfected with the defective hepatitis delta virus (HDV) the small HDV-RNA genomes borrow subviral HBsAg particles as outer coats to form the 36 nm circulating HDV virions [3]–[4]. Upon replication mediated by human RNA polymerase II (Pol II) HDV-RNA is released from hepacytes together with delta antigen (HDAg) as ribonucleoprotein complex (RNP) within the HBsAg envelope. The same human Pol II is involved into the synthetic pathway of microRNAs (miRNAs), a class of important regulatory elements of cellular epigenetics which are incorporated into specific RNP, RNA-induced silencing complex (RISC) [5]. MiRNAs have been increasingly implicated also into intercellular communications, as they were detected in serum either as free circulating RNA-induced silencing complexes (RISC) or in association with cell-derived particulate forms including exosomes and microvescicles [6]–[7]. Such a similarity of the RNP-related biological pathways in both HBV/HDV system and host cells prompted us to investigate whether HBsAg particles could provide housing to hepatocellular miRNAs for their release from HBV infected cells and circulation into the blood. In this study we report the isolation of the circulating HBsAg fraction from sera of 11 HBV carriers for full human miRNA profiling by real time quantitative PCR. Specific repertoires of hepatocellular miRNAs were found to be significantly associated with immunoprecipitated HBsAg.

## Materials and Methods

### Isolation of circulating HBsAg particles

**Figure 1:**
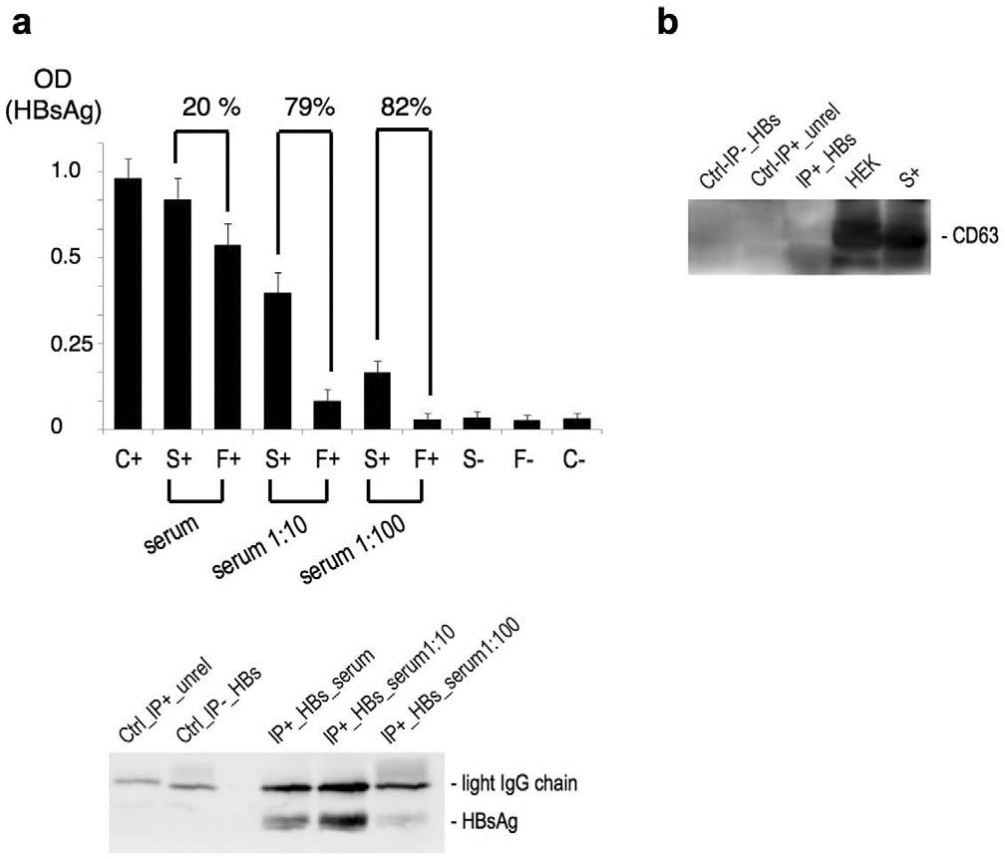
Isolation of serum HBsAg particles by immunoprecipitation. (a) Defining best immunoprecipitation yields. Upper panel: HBs-ELISA (BioRad) was used for measuring the depletion of HBsAg from sera under different HBs-immunoprecipitation conditions: antibody-to-resin ratio of 5 mg of antibody every 200 µl of sepharose-protein G slurry was kept since it proved to be the best working condition (not shown), while different starting dilutions of sera were screened. Depletion was calculated as % ratio of serum HBsAg OD values before and after each HBs-immunoprecipitation. Error bars indicate SD. C+, C−: positive and negative controls provided within the kit and used as for manufacturer's protocol. Bottom panel: qualitative rendering of HBsAg yields was obtained by Western blot analysis of HBs-immunoprecipitated fractions, followed by hybridization with mouse monoclonal anti-HBs antibody. (b) The exclusion of concomitant exosomes capture was evidenced by Western blot analysis of HBs-immunoprecipitated fractions with rabbit anti-CD63 antibody; HEK whole-cell lysate. Panels a-b are descriptive of at least three independent experiments.

HBsAg particles were immunoprecipitated with anti-HBs-IgG from 11 sera of HBsAg carriers (phase of HBV infection and disease was classified as previously reported [8] and 2 HBsAg negative controls), whose characteristics are described in Table 1. Sera were pre-cleared by incubation with sepharose-protein G slurry (GE Healthcare, UK; 120 min at room temperature), recovered by centrifugation and incubated overnight at 4°C with preformed sepharose-protein G-IgG complex for either a) HBsAg immunoprecipitation (where IgG = mouse monoclonal anti-HBs antibody, Santa-Cruz Biotechnologies, clone 1023), or b) control immunoprecipitation (where IgG = mouse monoclonal anti-human c-myc antibody, Invitrogen, clone 9E10.3) (Figure 1). After centrifugation we obtained leftover sera or flowthroughs and immunoprecipitated fractions for both HBsAg positive (n = 11) and HBsAg negative (n = 2) samples. In parallel, a control immunoprecipitation was performed on precleared sera of HBsAg negative controls by incubation with the same mouse anti-HBs-antibody, providing the corresponding flowthroughs (control anti-HBs-F, n = 2) and immunoprecipitated fractions (control anti-HBs-IP, n = 2). Best immunoprecipitation conditions for the isolation of circulating HBsAg were obtained by comparing different antibody-to-resin ratios (from 1 µg to 10 µg of antibody every 200 mL of sepharose-protein G slurry) as well as different starting dilutions of sera (undiluted, 1∶10 with PBS pH 7.4, 1∶100 with PBS pH 7.4).

**Table 1:** Demographic and Virologic Characteristics of Individuals and Sera.

Number	Age	Sex	HBV Genotype	HBV Infection Phase	HBsAg IU/ml	HBV-DNA IU/ml	ALT U/l	VP	SVP	SVP/VP
1	32	female	D	AC2	5459	1.62E+06	288	3.24E+08	4.80E+12	1.48E+04
2	38	male	D	AC2	12	6.80E+01	32	1.36E+04	1.06E+10	7.76E+05
3	45	male	A	AC2	4774	2.76E+05	53	5.52E+07	4.20E+12	7.61E+04
4	52	male	F	AC2	38565	1.50E+03	38	3.00E+05	3.39E+13	1.13E+08
5	49	male		CTRL Neg			73			
6	55	male		CTRL Neg			47			
7	40	female	D	IC	32.17	8.66E+02	10	1.73E+05	2.83E+10	1.63E+05
8	27	male	D	IC	0.355	5.20E+01	26	1.04E+04	3.12E+08	3.00E+04
9	35	male	D	AC1	1120	1.09E+03	22	2.17E+05	9.86E+11	4.53E+06
10	47	female	D	AC1	3955	1.71E+04	42	3.43E+06	3.48E+12	1.02E+06
11	43	female	D	AC2	26784	<12	24		2.36E+13	
12	51	male	D	AC2	5394	<12	28		4.75E+12	
13	44	male	D	AC2	10673	3.37E+04	43	6.74E+06	9.39E+12	1.39E+06

Abbreviations: VP = viral particles, virion; SVP = subviral HBsAg particles; IU = International Units; ng = nanograms; ALT = serum alanine amino trasferase.

Quantitative values for VP and SVP were obtained as previously reported [14]–[15]: 1 IU of HBsAg corresponds to 1,1E+06 IU HBV DNA and1 ng of HBsAg corresponds to 2,08E+08 SVP or 5,0E+07 VP.

HBV infection and disease phases were characterized as previuosly reported [8]: IC = Inactive HBsAg Carriers with serum HBV-DNA persistently below 2000 IU and without liver disease; AC1 = Active HBsAg Carriers with serum HBV-DNA fluctuating below 20.000 IU with normal liver histology; AC2 = Active HBsAg carriers with serum HBV-DNA fluctuating above 20.000 IU with chronic active hepatitis at histology, patients with chronic hepatitis B (CHB).

An alternative HBsAg immunoprecipitation was performed in all HBsAg positive sera using Invitrogen Immunoprecipitation Kit-Dynabeads® Protein G. Briefly the same mouse monoclonal anti-HBs antibody (10 µg) was crosslinked to 2,8 µm superparamagnetic beads (50 µl \30 mg/ml) using the cross-linking reagent BS3 (5 mM) and activated beads were used for the HBsAg immunoprecipitation according to the manufacturer's specifications.

### Ethics Statement

The study was approved by the Ethical Committee of the University Hospital of Pisa and partecipants gave their written consent.

### Analysis of immunoprecipitated HBsAg by Elisa and Western blotting

Samples were diluted 1∶300 with PBS and tested by commercial HBsAg ELISA (Bio-Rad). Yields of serum HBsAg depletion were then calculated as % ratio between OD values of starting sera and corresponding leftovers. Results were confirmed by quantitative Architect HBsAg assay (Abbott Laboratories) [8]. For each immunoprecipitation condition, all samples were analyzed either individually or pooled together.

Western blotting analysis was performed on all samples or on pooled samples, in order to evaluate possible serum contaminants by exosomes. In particular, protein lysates were prepared in 1% SDS-lysis buffer supplemented with protease inhibitors. Lysates were mixed with 1 volume of 2× SDS sample buffer, denatured, loaded on 12%SDS-PAGE, and blotted onto nitrocellulose filters (Millipore, Italy). Filters were hybridized with one of the following primary antibodies: mouse monoclonal anti-HBs (Santa-Cruz Biotechnologies, clone 1023), rabbit polyclonal anti-CD63 (Santa-Cruz Biotechnologies, clone H-193) or rabbit monoclonal (Cell Signaling, clone C34C6) anti-Ago2. This was followed by hybridization with secondary anti-mouse or anti-rabbit HRP-conjugated antibody (GE Healthcare, UK), and detection of chemiluminescent reaction (ECL plus, Amersham, GE Healthcare, UK).

Serial 1\10 dilutions (in PBS with an without 0.5% NP40 detergent) of the immunoprecipited fractions (IP) from HBsAg positive and negative sera were tested for Ago2 protein using the 96-well microplates of the HBsAg Elisa Kit (DIA.PRO Diagnostic Probes Srl. Milan, Italy) coated with monoclonal anti-HBs antibody.

The assay was carried out according to the protocols of the manufacturer's taking care to maintain the same incubation time for all the samples in testing. IP sample dilutions were incubated for 120 min at 37°C and after washing additional incubations for 60 min at 37°C were performed respectively for rabbit anti-Ago2 HRP conjugated antibody and rabbit anti-CD63 HRP conjugated antibody, used as control. After the cromogen\substrate incubation optical density was measured using a calibrated microplate reader equipped with a 450 nm filter and a second filter (620–630 nm) for banking and the S\N 2.1 ratio as positive\negative cut-off.

### Detection of host cellular miRNAs by RT-qPCR

Total RNA was isolated from all samples (whole HBsAg positive sera, n = 11; whole HBsAg negative sera, n = 2; HBsAg positive flowthroughs, n = 11; HBsAg negative flowthroughs, n = 2; HBsAg positive IP, n = 11;HBsAg negative IP, n = 2; control anti-HBs-F, n2 and control anti-HBs-IP, n = 2) by the mirVana miRNA Isolation kit (Ambion). After treatment with the mirVana miRNA Isolation kit lysis buffer, samples were added with 3.5 femtomolar *spike-in* miRNA (ath-miR-159a *Arabidopsis thaliana*, Applied Biosystems) before RNA isolation following the manufacturer's protocol. The quantity and quality of RNA samples were assessed either by spectrophotometer at OD_260/280_ (NanoDrop Technologies Inc.) or with Quant-iT RiboGreen RNA assay (Invitrogen). Profiling of miRNAs was carried out using TaqMan-based real time quantitative PCR (RT-qPCR; all reagents from Applied Biosystems) as previously described [9]. The manufacturer's Megaplex protocol with pre-amplification reaction was used; we obtained a good correlation between C_T_ values with and without the pre-amplification step and a very good reproducibility (data not shown). Pre-amplified products of reverse transcription were finally mounted on 384 microfluidic cards containing the TaqMan Low Density Arrays (TaqMan Gene Expression assays for 664 human miRNAs accordingly to mirBase v.10.1, also including duplicate assay for ath-miR-159a) for amplification. RT-qPCR reactions were run on ABI Prism 7900HT equipped with SDS software v2.3. Screening of miRNAs in control immunoprecipitates (obtained either with the mouse monoclonal anti-HBs antibody or mouse monoclonal anti-human c-myc antibody) was used to determine aspecifically immunoprecipitated serum miRNAs for the comparative ΔΔC_T_ analysis of data.

Quantification of miR122 levels was performed in duplicate on pellets and sera of HBsAg positive and negative subjects by TaqMan qRT-PCR assay on an Applied BioSystems 7300 HT thermocycler as previously reported^10^ using miR-181a and miR 182c as internal references [10]–[11]; specific primers were designed according to the miRBase data base (http://microrna.samger.ac.uk/). The miRNA levels were calculated according to ΔC_T_ values where ΔC_T_ = mean (Ct of internal reference)−Ct of target miRNA.

### Data analysis, statistics and bioinformatics

Raw C_T_ values were calculated using the SDS software v.2 and exported as “Amplification Data” text files for each sample. Exported data were processed with Excel. The global raw C_T_ values are available in a matrix format as tab delimited text file. As long as each miRNA in each sample was considered as detected only for values of C_T_<35 [9], [12]–[13], a downstream ceiling procedure was performed according to which all values C_T_≥35 were replaced with the chosen threshold of C_T_ = 35. Normalization was performed against *spike-in* ath-miR-159a: the mean of ath-miR-159a amplifications (two for each sample loaded and amplified on the 384 microfluidic card) was used as the computed control, and the change in cycling threshold was calculated for each miRNA (ΔC_T_ = C_T_miRNA_−C_T_ath_).

Both statistical analyses (MeV software version 4.6; unsupervised hierarchical clustering, unsupervised non-parametric Mann-Withney test, supervised gene distance matrix correlation and data rendering by single-gradient heatmaps) and the comparative ΔΔC_T_ calculations were generated from ΔC_T_ values. In particular, unsupervised hierarchical clustering either on the whole miRNA dataset or on significant miRNAs was performed by using the uncentered Pearson correlation coefficient and complete linkage. When comparing two groups, a non-parametric Mann-Whitney test was used to rank differentially expressed miRNAs, as required by data normality. For all tests, a p<0.05 was considered significant.

**Figure 2:**
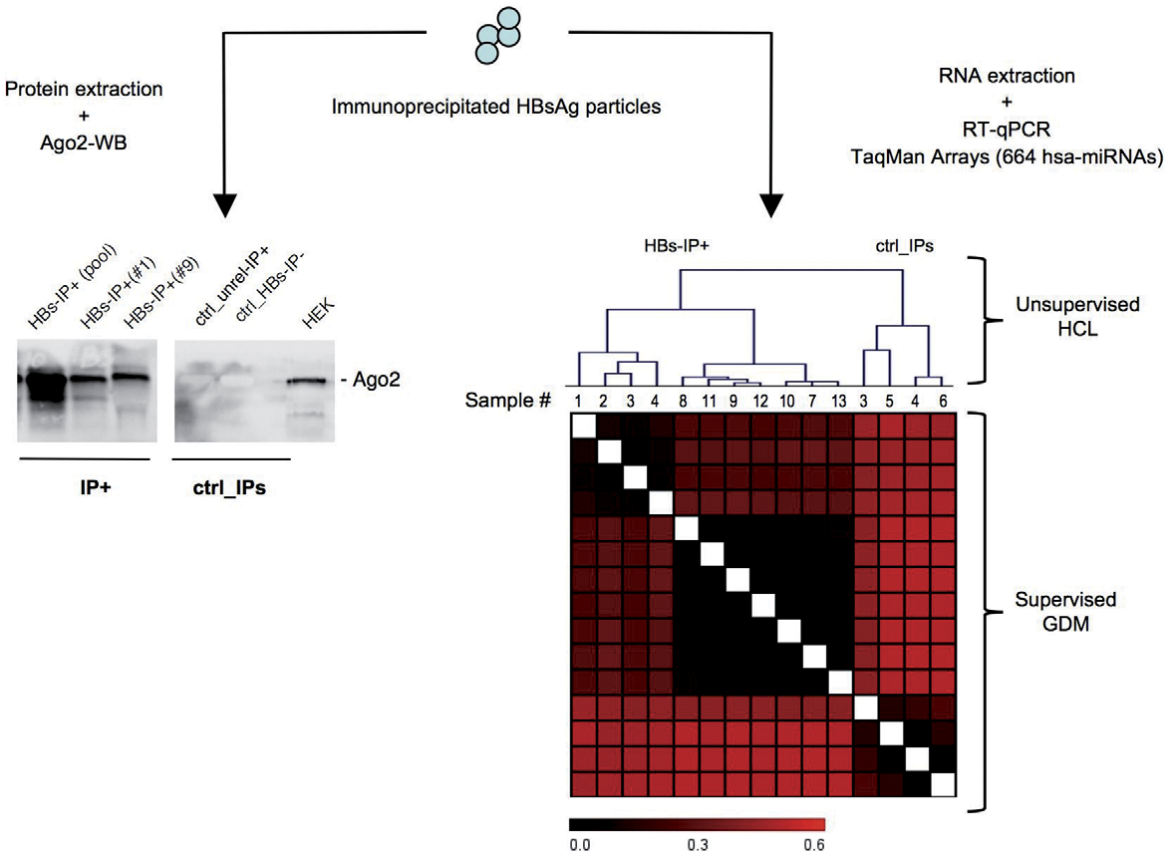
Differences in Western Blotting and human miRNA analyses between immunoprecipitated HBsAg particles and control immunoprecipitations. Left-most: Western blotting analysis of protein lysates from HBsAg positive immunoprecipitates (HBs-IP+) and control HBsAg negative immunoprecipitates (ctrl-IPs) for detection of Ago2 protein. IP+ lanes contain pooled protein lysates from samples # 1–13 and single lysates from samples # 1 and 9 respectively. Ctrl-IPs lanes contain protein lysates from immunoprecipitates obtained from HBsAg positive sera with mouse monoclonal anti-human c-myc antibody (unrelated-IP+) and HBsAg negative sera using anti-HBs monoclonal antibody (HBs-IP−). HEK is the protein lysate from HEK cells used as positive control for Ago2 protein. The figure is representative of 3 independent experiments. Right-most: HCL is unsupervised hierarchical cluster analysis of detected miRNAs (DC_T_ values) in both HBs-immunoprecipitated fractions from HBsAg positive immunoprecipitates (HBs-IP+) and control HBsAg negative immunoprecipitates (ctrl-IPs). GDM is supervised gene distance matrix correlating DCt values of HBs-IP+ vs ctrl-IP samples.

The pools of miRNAs associated with immunoprecipitated HBsAg were determined by the comparative ΔΔC_T_ method as in the following algorithm: a) since all control immunoprecipitation samples clustered together and were clearly separated from the HBs-IP+ samples (Figure 2, right-most), the whole group of control immunoprecipitation samples was used as the calibrator subset (control immunoprecipitates obtained either with the mouse monoclonal anti-HBs antibody or mouse monoclonal anti-human c-myc antibody); b) for each miRNA gene, the average normalized expression value (mean ΔC_T_) was calculated across the calibrator subset (overall n = 4); c) the HBsAg positive pellets (n = 11) were considered as target subsets, and ΔC_T_ values in each sample were compared to average ΔC_T_ values of the calibrator subset to generate ΔΔC_T_ values; d) ΔΔC_T_ values were then used to calculate the relative quantities (RQ = 2^−ΔΔCt^) between the calibrator group and the target subsets. For rendering reasons, RQ values were rescaled in a logarithmic scale (base 10); e) we choose a cutoff of 0.5 Log(RQ) corresponding to 3.16 RQ, so that only RQs above this threshold were taken into account; these selected RQs were used for studying the distribution of HBsAg-associated miRNAs among different hepatitis B infection and disease phases.

When the comparative ΔΔC_T_ analysis was aimed at defining the circulating HBsAg-miRNA signature, we made further selection on miRNAs as follows: a) only those miRNAs with RQs that were successfully selected as described above in at least 2 of the profiled HBsAg positive IP samples were further considered; b) next, average RQs of such selected miRNAs were calculated across the target HBsAg positive IP samples along with SD and represented as rescaled logarithmic base 10 plot.

A further refinement of the overall circulating HBsAg-miRNA signature was made by selecting miRNAs with a non-parametric Mann-Whitney test (p<0.05) on the average C_T_ values calculated across the target HBsAg positive IP samples versus the average ΔC_T_ values calculated across the calibrator control HBsAg negative immunoprecipitates.

### Targets prediction of HBsAg-associated miRNAs and functional pathway analysis

Targets prediction was performed for each HBsAg-associated miRNA of the overall circulating HBsAg-miRNA signature by TargetScanHuman, release v5.1 (http://www.targetscan.org), taking into account only conserved target sites. Predicted genes were then ranked on the basis of a combined parameter (multiplicative value) obtained by multiplying the TargetScan total context score by the number of conserved sites. To avoid exceeding the number of both gene list and predicted pathways, only targets with multiplicative values ≤−1 were selected and used as input list for functional pathway exploration by Ingenuity Pathway Analysis (IPA) software (Ingenuity Systems, CA). IPA-generated top-ranked pathways were determined on the basis of the incidence of predicted miRNA targets in a list of canonical pathways provided by the IPA software. Canonical pathways thus obtained were ranked according to their significance in a Fisher exact test (p<0.05).

## Results

Quantitative purification of HBsAg particles from sera of HBV carriers was performed by selective immunoprecipitation with mouse monoclonal anti-HBs antibody to efficiently adjust experimental conditions and gain the highest reproducible HBsAg yields. In fact, as long as the isolation of HBsAg fraction from HBsAg positive samples was aimed at microRNA (miRNA) profiling studies, high yields were important to meet sensitivity of real time quantitative PCR (RT-qPCR). To this aim, sepharose-protein G resin was employed as solid phase for the immunoprecipitation reaction because of its high IgG binding capacity, which allowed for both efficient elimination of IgG contaminants from sera as well as quantitative depletion of HBsAg from sera. Best immunoprecipitation conditions were obtained by comparing different antibody-to-resin ratios (from 1 µg to 10 µg of antibody every 200 µl of sepharose-protein G slurry) as well as different starting dilutions of sera (undiluted, 1∶10 with PBS pH 7.4, 1∶100 with PBS pH 7.4). In order to evaluate best quantitative yields and determine reproducibility of HBsAg recovery from sera, we measured serum HBsAg depletion before and after each immunoprecipitation by a commercial HBsAg-specific Elisa. For each immunoprecipitation condition considered, analysis was taken on both individual and pooled samples. As long as we found that best working antibody-to-resin ratio was 5 µg of antibody every 200 µl of sepharose-protein G slurry (not shown), we kept this parameter for further fine tuning of the immunoprecipitation conditions (Figure 1a, upper panel). In parallel, HBsAg isolation yields were evaluated by Western blotting of the immunoprecipitated fractions (HBsAg positive IP samples; Figure 1a, bottom panel), and compared with HBsAg depletion yields from sera (Elisa). Elisa and Western blot results unanimously indicated that in our hands the preparative immunoprecipitation conditions assuring for optimal balance between highest ratios of HBsAg depletion from sera (ca 79%) and absolute quantitative yields of HBsAg into immunoprecipitated fractions were the following: serum diluted 1∶10 with PBS, 5 µg of antibody every 200 µl of sepharose-protein G slurry.

Immunoprecipitation conditions are critical to avoid miRNA contaminations from other components of sera (principally exosomes and circulating immunocomplexes which represent main circulating carriers of human miRNAs in sera), and purity of HBsAg preparations was as important as quantitative high yields. Therefore, in setting the HBsAg isolation conditions, a series of control immunoprecipitations were also undertaken to assess possible serum contaminants and improve specificity. Western blotting analysis indicated that HBsAg protein were not present into the immunoprecipitated fractions of control IPs (Figure 1a, bottom panel), thus evidencing that both the anti-HBs antibody used and the selected isolation conditions granted for specific obtainment of the HBsAg fraction. A consistent evidence of the lack of exosome contamination was obtained by Western blotting analysis: nor the enriched HBsAg fractions neither the control IPs presented any protein signal due to classic exosomal marker CD63 (Figure 1b).

We looked for the presence of RISC-associated Argonaute2 (Ago2) protein into the immunoprecipitated fractions by Western blotting, and we found that Ago2 was associated with HBsAg positive IP (Figure 2, left-most). Using an Elisa with a solid phase coated with the monoclonal anti-HBs antibody we could reveal the Ago2 protein with rabbit anti-Ago2 HRP conjugated antibody only after NP40 treatment (up to 1\1000 dilution in sera 1and 3 which have the highest serum HBsAg levels (Table 1), but not without NP-40 treatment. Negative results were obtained using rabbit anti-CD63 HRP conjugated antibody as second antibody.

Based on these overall results, the best HBsAg isolation condition (serum diluted 1∶10 with PBS, 5 µg of antibody every 200 µl of sepharose-protein G slurry) was used throughout the whole study as it provided highest HBsAg immunoprecipitation yields, optimal specificity, as well as purity.

To evaluate the presence of human miRNAs within immunoprecipitated HBsAg fractions, we used RT-qPCR methodology associated with TaqMan miRNA Arrays which encompass 664 mature miRNAs from the Sanger mirBase v.10.1. The whole repertoire of human miRNAs was screened in each sample by RT-qPCR to obtain raw cycle-threshold (C_T_) expression values. Raw C_T_ data were then computationally processed as described in Materials and Methods. For normalization and comparison of data among individual samples, we used the *spike-in* ath-miR-159a as internal standard. The choice was compelled by our experimental system (virus-related particles), which rendered impractical the use of established human endogenous/housekeeping genes (e.g., 18S rRNA, GAPDH, ACTB, or small noncoding miRNAs such as RNU48, RNU44, U6, U18, and U47), as well as by the fact that to date no established viral miRNA gene from HBV has been assayed and measured. Moreover, the use of HBV-DNA amplification by RT-qPCR was considered inefficient because a) the HBs-immunoprecipitation involved all the repertoire of HBsAg-positive circulating particles, namely virions and defective particles, with great variability of the two fractions from sample to sample; b) viral load (HBV-DNA serum levels) varies in relation to HBV infection and disease phase. On the contrary, the exogenous standard ath-miR-159a assured for technical normalization while being independent of biological variability, and its use as normalization factor was validated in our hands by convergent results with the global mean approach (not shown) which has recently been accepted for miRNA profiling of biological systems lacking endogenous controls.

When analyzing normalized expression values (ΔC_T_) for unsupervised hierarchical clustering (HCL, Figure 2, right-most), all control IP samples clustered together and were clearly separated from the group of HBsAg positive IP samples. This fact was well evidenced also by graphical visualization of the whole ΔC_T_ dataset as a supervised gene distance correlation matrix (GDM) built up on the result of the HCL analysis. These observations indicated the presence of human miRNAs specifically associated with immunoprecipitated HBsAg particles and prompted us to characterize miRNA profiling.

**Figure 3:**
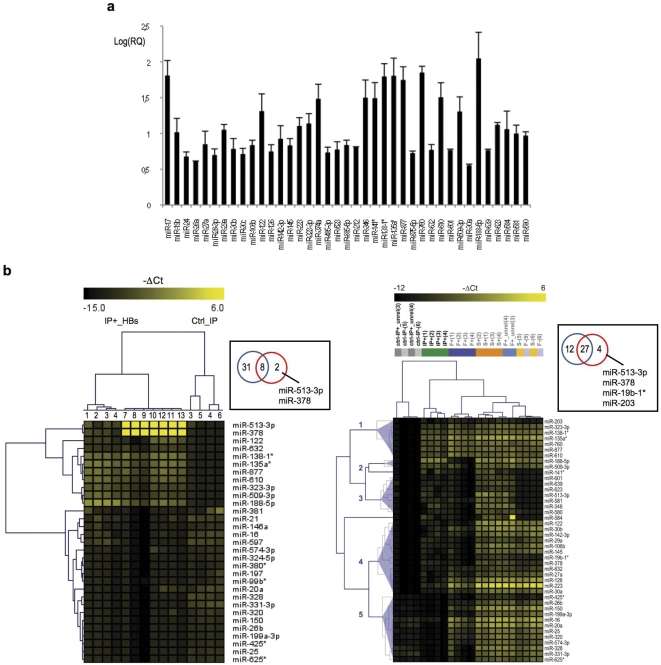
Identification of human miRNAs associated with serum HBsAg. (a) The circulating HBsAg-miRNA signature: average RQs were obtained from the comparative DDC_T_ analysis. Values are reported in a bar plot as a logarithmic scale base 10 along with SD. (b) Differentially detected miRNAs between HBsAg positive immunoprecipitates (HBs-IP+) samples (left-most, n = 11; right-most, n = 4) and the group of control HBsAg negative immunoprecipiates (ctrl-IPs) (n = 4) were selected by Mann-Whitney test on –DCt values (left-most, p<0.1; right-most, p<0.05), and an unsupervised hierarchical cluster analysis was finally performed. Venn diagrams indicate the comparison among the pool of HBsAg-associated miRNAs obtained from the comparative DDC_T_ analysis of panel a (blu circle) and the HBsAg-associated miRNAs obtained from the Mann-Whitney tests (red circle).

In the next step, a comparative ΔΔC_T_ analysis was perfomed to identify the pools of miRNAs associated with the immunoprecipitated HBsAg particles, as described in Materials and Methods. Since all ctrl-IP samples significantly clustered together (Figure 2, right-most), the whole group of control IPs was used as the calibrator subset. On the other hand, the HBsAg positive IP samples (n = 11) were considered as target subsets, and ΔC_T_ values in each HBsAg positive IP sample were compared to average ΔC_T_ values of the calibrator subset, in order to obtain relative quantity (RQ) values which describe the ratio of miRNA average expression levels between each target HBsAg positive IP sample and the calibrator subset. Only RQs whose value overwhelmed a fixed threshold of 3.16 (corresponding to a more than three-fold expression level in each HBsAg positive IP sample with respect to control IPs) were further selected. As further stringency in the comparative DDC_T_ analysis, we considered only those miRNAs with successfully selected RQs in at least 2 of the profiled HBsAg positive IP samples. Finally, average RQs of thereby selected miRNAs were calculated across the target HBsAg positive IP samples along with SD, and represented as rescaled logarithmic base 10 plot (Figure 3a). Collectively, these HBsAg-associated miRNAs were used throughout the study as the circulating HBsAg-miRNA signature.

In order to verify the pool of HBsAg-associated human miRNAs identified by the comparative ΔΔC_T_ analysis described so far, a different approach was used in which the individual ΔC_T_ values of each HBsAg positive IP sample were directly compared to individual DC_T_ values of each control IP sample within a two experimental groups, non-parametric Mann-Whitney test followed by hierarchical clustering on significant genes. The analysis was aimed at determining those miRNAs with significant differentiated detection levels between the two groups, and it was first performed by engaging all of the HBsAg positive IP samples. As shown in Figure 3b (left panel) the Mann-Whitney test led to a pool of 11 miRNAs that were significantly associated to the immunoprecipitated HBsAg particles and were all contained within the group of HBsAg-associated human miRNAs identified by the comparative ΔΔC_T_ analysis (Figure 3a) with the exception of miR-513-3p and miR-378 (Venn diagram in the Figure 3b, left panel). In fact these 2 miRNAs presented a great SD because of the strongly heterogeneous level distribution among samples, leading to their exclusion from the comparative ΔΔC_T_ analysis of Figure 3a.

**Figure 4:**
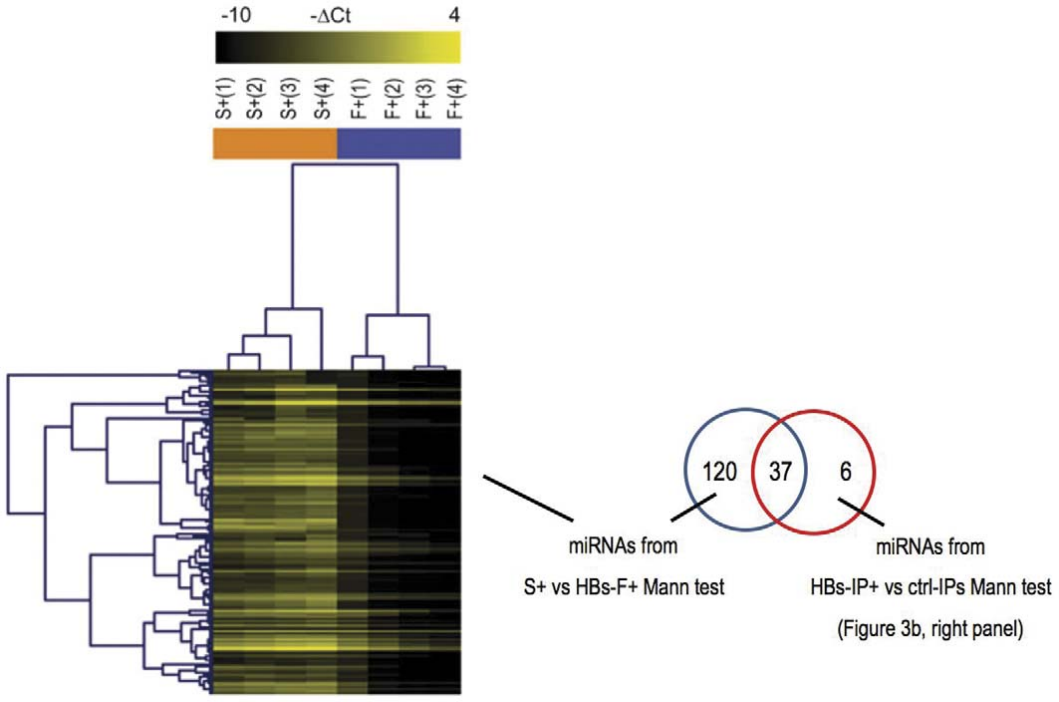
HBs-immunoprecipitation led to a significant change of detection of some human miRNAs in examined sera. Heatmap of differentially detected miRNAs in whole HBsAg sera (S+) and HBsAg positive flowthroughs after immunoprecipitation (HBs-F+) was obtained by Mann-Whitney test (p<0.05) followed by hierarchical clustering (-DC_T_ are represented). Venn diagram: the 157 differentially abundant serum miRNAs between whole HBsAg positive sera (S+) and HBsAg positive flowthroughs (HBs-F+) were compared to miRNAs of clusters 1-to-5 in Figure 3b (right panel).

Given the heterogeneity of the examined samples belonging to different HBV infection and disease phases, the two experimental groups, non-parametric Mann-Whitney test was then repeated on a smaller group of HBsAg positive IP samples, belonging to the AC2 phase of HBV infection (Table 1), namely patients with chronic hepatitis B (CHB) (Figure 3b, right panel). As expected, the reduction of the heterogeneity of samples produced a larger number of miRNAs significantly associated with immunoprecipitated HBsAg. All of the identified miRNAs belonged to the circulating HBsAg-miRNA signature defined in the comparative ΔΔC_T_ analysis of Figure 3a, with the exception of a few HBsAg-associated miRNAs which were negatively selected by the ΔΔC_T_ analysis because of high SD values (Venn diagram in the Figure 3b, left panel). In this second Mann-Whitney analysis, the differentiated repertoires of human miRNAs that significantly associated with the immunoprecipitated HBsAg particles were organized into clusters 1–4. Aspecifically immunoprecipitated miRNAs were grouped in cluster 5. In particular, HBsAg-associated miRNAs in clusters 2–4 were characterized by a significant decrease of their serum levels upon the HBs-immunoprecipitation. Following this latter observation, a Mann-Whitney test was also performed on normalized ΔC_T_ values of whole HBsAg positive sera and HBsAg positive flowthroughs from the examined samples # 1–4 of the AC2 phase of HBV infection, CHB (Table 1). The heatmap of differentially detected miRNAs (Figure 4) clearly shows that a number of serum miRNAs (including the identified HBsAg-associated miRNAs of Figure 3) underwent a significant change-fold of detected levels upon the HBs-immunoprecipitation. In particular, when comparing by Venn analysis the 157 differentially abundant serum miRNAs between whole HBsAg positive sera and HBsAg positive flowthroughs to the miRNAs obtained by the Mann-Whitney test of Figure 2s (right panel, clusters 1-to-5), it was observed that miRNAs from clusters 2–4 fall in the set of the 37 common miRNAs (Venn diagram of Figure 3b), thus contributing to the observed serum differences upon HBs-immunoprecipitation, the rest being represented by aspecifically immunoprecipitated miRNAs of cluster 5. To analyse the quantitative relations between hepatocellular miRNA and HBsAg we measured the levels of the most frequent and more abundant liver specific miRNA, miR-122 by qRT-PCR and found that median miR122 levels were significantly (p<0.001) higher in pellets (42.1 in HBsAg positive and 0.19 in HBsAg negative samples) vs whole HBsAg positive and negative sera respectively (0.41 and 0.05) showing a significant enrichment (>100 fold) of miR-122 in HBsAg positive pellets vs whole sera and about 20 fold increase in HBsAg positive vs HBsAg negative pellets.

**Figure 5:**
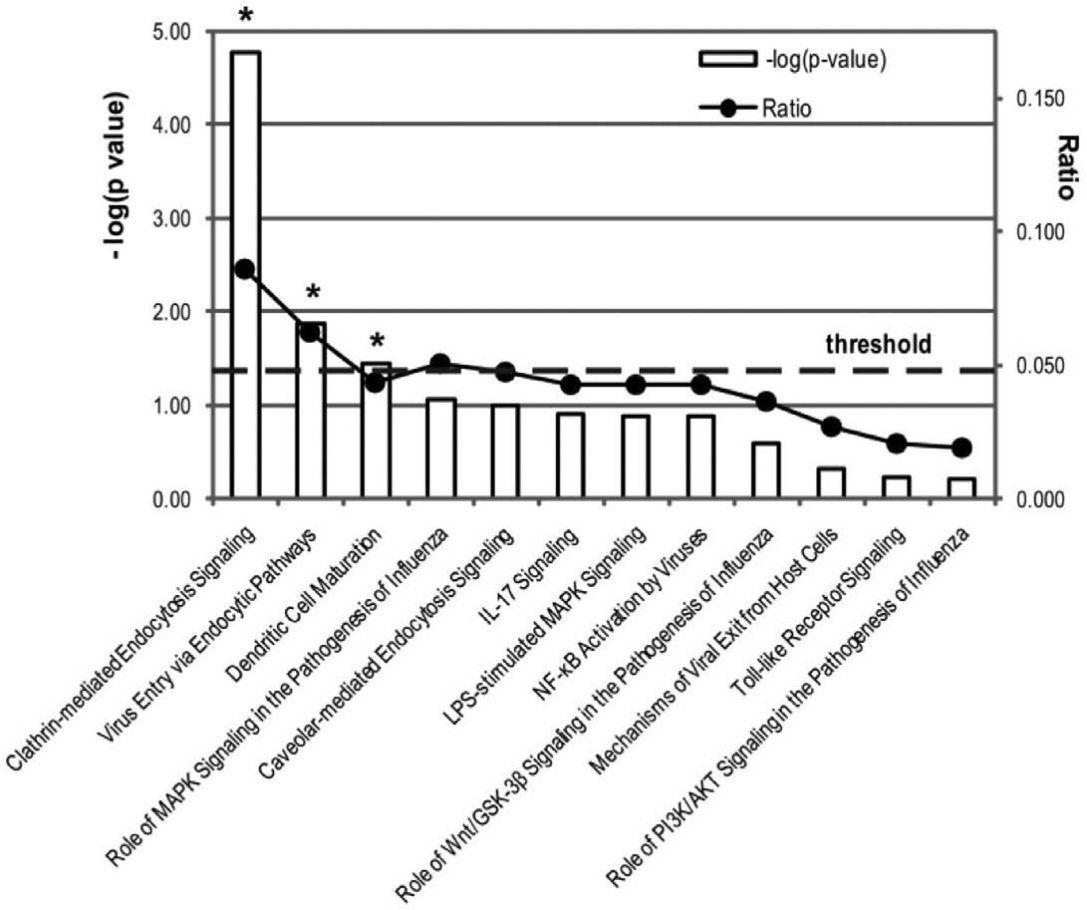
Canonical pathways enriched by predicted targets of HBsAg-associated miRNAs. Target genes of HBsAg-associated miRNAs (Figure 3a) were predicted (TargetScan) and ranked. Functional analysis (IPA) was then applied on predicted target genes, and canonical pathways were finally ranked according to their significance in a Fisher exact test (p-value≤0.05, horizontal threshold line in the plot). Left axis: –log(p-values) as histogram boxes; the asterisk (*) marks the three pathways that resulted as significantly enriched by the analyzed gene-list; right axis: for each pathway, black dots indicate the ratio of pathway-defining genes, which is calculated as the number of involved target genes over the total number of genes in that pathway reference dataset.

We assessed the putative target genes of these miRNAs by a well-established miRNA-target prediction software, TargetScan. The algorithm was used with default parameters, but in order to increase the stringency we selected miRNA-targets within a given threshold (Materials and Methods). Top-scoring miRNA-targets were then used as input list for a functional pathway analysis by Ingenuity Pathway Analysis software, a well-established tool for in silico evaluation of pathways involving candidate target genes. HBsAg-associated miRNAs and their putative targets were significantly linked to 3 canonical pathways dealing with clathrin-mediated endocytosis, virus entry via endocytosys and dendritic cell maturation (Figure 5).

## Discussion

In the present study we show for the first time that HBsAg particles circulating in HBV carriers carry hepatocellular miRNAs and the Ago2 associated protein. The immunoprecipitation of HBsAg from whole serum leads to significant quantitative changes between miRNA levels before and after HBsAg particle depletion whereas this does not occur after control immunoprecipitations.

One hundred and fifty seven miRNA decreased significantly after HBsAg immunoprecipitation (Mann-Whitney test p<0.05, Figure 4); 39 of them were HBsAg associated according to them DDCt comparative analysis (Figure 3a), 31 (Mann-Whitney test p<0.05) if considering only patients with chronic hepatitis B. Using for HBsAg immunoprecipitation the same mouse monoclonal anti-HBs antibody crosslinked to 2,8 µm superparamagnetic beads we obtained a good correspondence in the output of HBsAg associated miRNAs (32\39, 82.05%). These results indicate that HBsAg associated miRNAs are distinct from the overall serum miRNA profile.

The great majority of the hepatocytes of HBV carriers are infected and produce subviral HBsAg particles as well as complete virions, but subviral particles outnumber virions (between 10.000–1.000.000 times in our cases, see Table 1). Accordingly, we found that most of HBsAg-associated miRNAs (namely, miR-122, miR-27a, miR-29a, miR-30a, miR-30b, miR-126, miR-145, miR-223) belong to the previously described repertoires of miRNAs differentially expressed in liver development, disease, fibrosis and/or regeneration [16]–[22].

Capturing the circulating HBsAg particles with a solid phase coated with the same monoclonal anti-HBs antibody used for the immunoprecipitation we could demonstrate that Ago2 protein can be revealed by the second sandwich HRP labeled antibody after NP40 treatment, but not without the disruption of the lipoprotein envelope by detergent treatment. In addition negative results were obtained using the control anti-CD63 HRP labeled polyclonal antibody as second antibody. These results provide consistent evidence that subviral HBsAg particles can carry inside miRNA and associated Ago2 protein in the same way as it was previously shown for HDV-RNA and HDAg complexes [3]–[4].

By measuring the most frequent and abundant mi-122 by qRT-PCR we attempted to normalize the amount of miR-122 to the respective HBsAg levels and with the limitation of the simplified simulation we found that there is a direct relation between the overall amount of HBsAg and miRNA in the testing material. However, this issue will be more properly addressed only by studying the 3D ultrastructure of HBsAg subviral particles in the electron microscope. As far as the possible functional activity of HBsAg associated miRNA we may only speculate that their amount appear significantly higher than in the exosomal and microvescicular compartments of the same sera. Recently is has been shown that miRNA carried by exosomes are biologically active once exosomes are internalized within cells [23]. However, we believe that the most relevant finding of our work is the identification of a new tool for non invasive liver specific miRNAs profiling that allows to study in simple serum specimens the dynamic changes of hepatocellular miRNA expression profiles in both physiologic and pathologic conditions. Interestingly we found a 30% correspondence between our results and those of Ji F et al. [10] who studied the overall serum miRNA profile of HBV infected patients. Interestingly we detected 5 of 9 miRNA, more frequently circulating in their HBsAg carriers: miR-19b, miR-122 and miR-223 were associated with HBsAg positive immunoprecipitates whereas miR-16 and miR-20a were detected in control immunoprecipitates.

Different studies highlight the emerging role of miRNAs in host-pathogen interactions not only in plants and invertebrates, where miRNA-mediated RNA interference represents an innate antiviral immune response, but also in mammals [24]. Accordingly, liver-specific miR-122 was previously shown to be essential for hepatitis C virus replication [16], [25] and upregulated in HBV infected patients [26]. Some of HBsAg-associated miRNAs were reported to play a role in human carcinogenesis. Down-regulation of miR-122 and miR-223 was hyptohesized to be relevant for hepatocellular carcinoma development [18], [19], [27]. Recently Gao et al. [26] proposed miR-145 as a candidate tumor suppressive miRNA in the early steps of HBV related hepatocarcinogenesis.

In addition some of the HBsAg-associated miRNAs identified in the present study are directly involved in development/function of the myeloid lineage, as well as in the regulation of innate immunity (miR-223) and lymphocytes (miR-106b and miR-19b of the two mammalian paralog clusters miR-17∼92 and miR-106b∼25) [24].

On the basis of these considerations, the analysis of target genes of HBsAg-associated miRNAs and the related functional pathway analysis provided results which are strongly compatible with the biological activity of the identified miRNAs. In fact, computationally predicted target genes of HBsAg-associated miRNAs appeared significantly involved into molecular mechanisms canonically dealing with host-virus interaction, such as clathrin-mediated endocytosis, virus entry, and dendritic cell maturation. Interestingly, the finding that host-virus interaction pathways are over-represented among predicted targets of HBsAg associated-miRNAs suggests the intriguing hypothesis that circulating HBsAg particles carrying host cellular miRNAs might have an important role in the complex mechanisms of viral spreading, persistence and pathogenesis. This hypothesis is consistent with the evidence that HBsAg serum levels, resulting from the equilibrium between virus and host's immune system, vary in the different phases of HBV infection and reach the lowest values in the inactive HBsAg carriers [8], [28].The small number of cases of the present study does not allow any speculation on the role of different miRNAs, selectively associated with circulating HBsAg in the different phases of HBV infection and disease.

In conclusion, the finding that HBsAg particles carry hepatocellular miRNAs opens new avenues of research to disentangle the complex interaction between host and HBV. HBsAg associated-miRNAs might play a pivotal role in viral persistence and help to explain the clinical evidence that the most efficient immune control of HBV infection associates with the loss of serum HBsAg. Profiling the in vivo HBsAg-associated miRNAs dynamics in different cohorts of HBsAg carriers provides a unique model for studing the epigenetics of liver physiopathology in viral and non viral diseases.
